# Self-rated health and factors influencing responses among young Egyptian type 1 diabetes patients

**DOI:** 10.1186/1471-2458-11-216

**Published:** 2011-04-07

**Authors:** Heba Ismail

**Affiliations:** 1 Cairo University Specialized Children's Hospital, Cairo, Egypt

## Abstract

**Background:**

Patients diagnosed with type 1 diabetes mellitus (T1DM) face major daily challenges. Self-rated health (SRH) is a global measure of an individual's health related quality of life (HRQoL) and is based on the question, "In general, how would you rate your health?" Subjects rate their health as excellent, very good, good, poor or very poor. Our objective was to determine the HRQoL using the SRH measure and determine factors influencing responses. We hypothesized that better SRH responses were associated with shorter diabetes duration, better compliance and better glycemic control.

**Methods:**

The standardized SRH measure was the instrument used for health related quality of life assessment. Logistic regression analysis was used to examine the association between SRH responses and selected variables.

**Results:**

124 subjects, 64 females (51.6%) and 60 males (48.4%) were included. Average age was 13.08 (±3.19) and average diabetes duration was 5.82 (±1.60), while the mean HbA_1_C was 8.02 (±1.60). The majority rated their health as good (31%), 29% rated it as excellent, 11% as very good, 14% as poor and 15% as very poor. Regression analysis showed that regular exercise was the only predictor that was independently and significantly associated with a "better" self-health rating, with an OR of 12.84, CI of 1.425-115.727 and a *p *value of 0.023.

**Conclusion:**

Regular exercise among Egyptian children with T1DM is strongly associated with a "better" overall health related quality of life and should be repeatedly encouraged.

## Background

Diabetes mellitus affects nearly 3.9 million individuals in Egypt with an expected increase by 2025 to nearly 9 million [1]. This is a significant number, making health-related quality of life (HRQoL) among individuals with diabetes a public health goal.

Patients diagnosed with type 1 diabetes mellitus (T1DM) face major lifestyle changes and the risk of experiencing debilitating and life-threatening complications. The daily management of diabetes in and of itself presents numerous challenges to achieve adequate metabolic control; from multiple daily injections and frequent blood glucose monitoring to routine laboratory work, frequent healthcare visits, and careful regulation of exercise and meal schedules. Moreover, patients with T1DM are at increased risk for major depression, anxiety, and eating problems [2]. Considering T1DM is commonly diagnosed in children and young adults, the effect of these challenges become more pronounced as they accompany these young individuals for many decades to come. It is therefore essential that care for T1DM both alleviate the physical complications of the disease and improve overall health related quality of life.

The term "health-related quality of life" (HRQoL) has evolved to encompass the aspects of overall quality of life that are most clearly affected by either physical and/or mental health [3]. One way to measure health related quality of life is by employing self-reported measures of health. Self-reported health is often found to reflect several aspects of health including disease severity, aspects of positive health status, physiological and psychological reserves as well as social and mental functions [4-8].

One measure of self-reported health is the self-rated health (SRH) assessment tool. SRH is a global, self-assessment of an individual's current health status that has been used and validated since the 1950s [9]. It is one of the core measures widely recognized as a comprehensive indicator of HRQoL. Its comprehensiveness is often referred to as surprising because the indicator is based on a single question. In diabetes patient populations, it has been used extensively for HRQoL surveys [10-12]. It is based on the question, "In general, how would you rate your health?" or "In general, compared with others your age, how would you rate your health?" Subjects then rate their own health as excellent, very good, good, poor or very poor. Studies have shown that SRH reflects a complex process of internalized calculations that encompass both lived experience and knowledge of disease causes and consequences [13].

### Objective

We aimed to describe the SRH responses as a measure of HRQoL among younger Egyptian patients with T1DM. We also hypothesized that better SRH responses were associated with shorter diabetes duration, better compliance (including regular follow up, regular monitoring of blood sugars, intensive therapy, hospital visits and regular exercise) as well as better glycemic control.

## Methods

### Patient population and settings

At Cairo University Children's Hospital, we follow a large patient population with T1DM. Our patient population is of a low socioeconomic level. Treatment regimen for our patients is usually individualized, aiming to maintain normoglycemia as is possible. The majority of our patients follow an intensive regimen, defined as three or more insulin injections along with three or more blood glucose measurements per day [14]. Occasionally, due to poor compliance, glycemic control is maintained with a less-intensive regimen. Patients are also encouraged to exercise regularly as part of their diabetes management.

Patient visits are scheduled at least every 3 months and visits include an evaluation by a physician, as well as additional dietary evaluations by a registered dietitian during some visits. Glycohemoglobin (HbA_1_C), is a measure of glycemic control over the past three months, and is routinely done for our patients. Additional annual routine screening for diabetes complications include screening for microalbuminuria, liver and kidney function tests, lipid profile, as well as screening for associated autoimmune thyroid dysfunction.

Over a three months period, starting February of 2009 till May of 2009, subjects with T1DM aged 9 years and older presenting to our clinic for routine follow up were included in the study. The purpose of the study was explained to the patients and their parents, and an informed oral consent/assent was obtained. Subjects who were psychologically or mentally challenged were excluded, as well as subjects with diabetes duration < 1year. This study has been approved by the ethics committee at Cairo University in January of 2009.

### Data Collection

All subjects' charts were reviewed to obtain glycohemoglobin values (either ordered at the time of the visit or within the last 3 months) as well as documented number of acute events in the past 12 months preceding the study participation. Acute events were defined as emergency room visits or hospital admissions because of diabetes-related medical problems (eg. hypoglycemia or DKA). Data from the chart was also obtained to classify the intensity of the T1DM management regimen. Values for HbA_1_C were determined using fresh venous blood specimens. The nondiabetic range of our laboratory is 4.2-6.2%, and patients were advised that a value below 8% is considered acceptable blood glucose control.

Other data obtained from the patients' charts included: diabetes duration, presence of diabetes associations (hypertension, thyroid, celiac or other autoimmune problems), complications (nephropathy, retinopathy and neuropathy) as well as a family history of diabetes.

### Questionnaire

The standardized SRH measure was the instrument used for health related quality of life assessment. SRH as a measure of HRQoL has been widely used in diabetes patient populations, providing us with a rich repertoire of literature by which we can compare our population's HRQoL to [10-12]. The wording of the questionnaire was carefully chosen to be brief and easy for the children to understand. Colloquial Egyptian Arabic was used. We followed the method of Ajrouch and Moaddel [15] by translating the original English format into Arabic by professional medical Arabic translators, and then translating back into English and comparing with the original survey in order to ensure reliability. The SRH tool was scored such that positive readings received higher scores (from very poor = 1 to excellent = 5). Additionally, patients were asked if they exercised regularly or not (defined as at least 30-45 minutes at least 3 times a week). Moderate activity level in the form of housework, farm work or long walks for the aforementioned duration and frequency was also considered exercise.

### Statistical analysis

The analysis is in two steps. First we describe the SRH response among patients with T1DM. Second, we limit our analysis to the 'poorer' versus "better" response groups (after excluding those who responded "good"), and explore factors related to each of these two response groups.

Descriptive statistics were used in the form of mean, median, standard deviation and range as appropriate. After translating the patients' responses into scores, associations were attempted.

We were further interested in looking more closely at those who rated their health as either better than good (i.e. very good or excellent) and those who rated their health as poorer than good (i.e. poor and very poor) in an intensity sampling method [16] in order to determine predictor variables. In order to understand this better, we combined groups together. Those who responded very poor or poor were grouped together into the "poorer" response group, and those who responded very good or excellent were grouped together into the "better" response group. From this we created a dichotomous measure coded 0 if the response was "very poor" or "poor" (i.e. the "poorer" group) and 1 if the response was "very good" or excellent" (i.e. the "better" response group). In dichotomizing SRH in our analysis, we follow the lead of Manor et al [17] who observed that dichotomization of SRH response groups does not affect results.

Logistic regression analysis was used to evaluate the independent contribution of various predictor variables to the survey score results. Logistic regression models were generated so that, using self-rated health "poorer" and "better" response groups as the dichotomous dependent variable, we could ascertain which demographic and health-related variable(s) were independently associated. Independent variables examined included gender, patient age, HbA_1_C values, diabetes duration, regular follow-up, type of treatment regimen, self monitoring of blood glucose (SMBG), regular exercise, acute complications, chronic complications, associations, and family history. Odds ratio (OR), 95% confidence intervals (CI) and p values were used for comparison. Statistical significance was set at *p *< 0.05 (*p *values are two tailed). Tests for interaction effects and goodness of fit were also performed.

## Results

Our sample consisted of 124 subjects, 64 of which were females (51.6%) and 60 were males (48.4%). Mean patient age was 13.08 (± 3.19) with a median of 12.09 (range 9-25), while mean diabetes duration was 5.82 (±4.47) with a median of 4.11 (range 1-23). Regarding diabetes management, the average glycohemoglobin value was 8.02 (±1.60) while the median was 7.9 (range 5.4-12.6). Table 1 describes subjects' characteristics.

**Table 1:** Subject Characteristics

	**No**.	%
**Gender**		
• **Male**	**60**	**48.4**
• **Female**	**64**	**51.6**
**Regular clinic follow up (every 1-3 months)**	**96**	**77.4**
**Intensive therapy (≥3 shots/day)**	**108**	**87.1**
**SMBG (≥3 times/day)**	**110**	**88.7**
**Regular exercise**	**30**	**24.2**
**Acute complications**	**58**	**46.8**
**Associated conditions**	**24**	**19.4**
**Chronic complications**	**62**	**50**
**Positive family history**	**78**	**62.9**

SMBG: self monitoring of blood glucose

**Figure 1:**
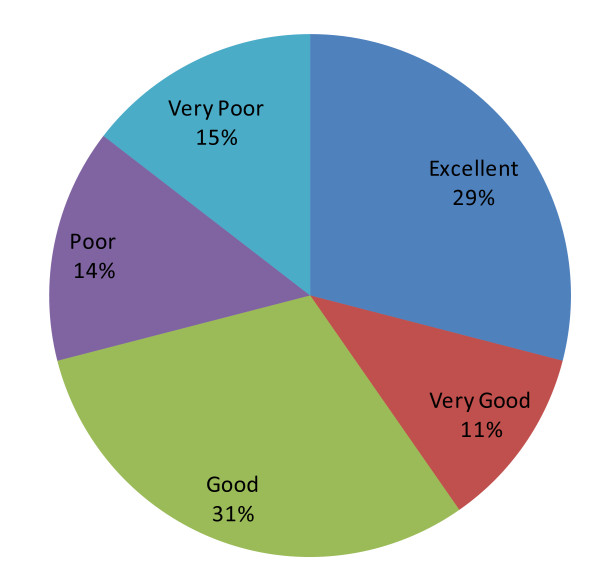
**SRH response groups**.

Figure 1 illustrates subjects' SRH responses by percentage, with the majority of subjects (n = 38, 30.7%) rating their health as "good".

The majority of patients followed an intensive management regimen with only 12.9% (n = 16) taking 2 shots/day (pre-mixed insulin) and 11.3% (n = 14) testing their BG <3 times per day. Thirty subjects (24.20%) reported exercising regularly at least three times per week. Also, the majority (77.4%, n = 96) followed up regularly at the clinic. Fifty eight subjects (46.80%) reported acute diabetes related complications requiring either hospitalization or an ER visit (severe hypoglycemia or DKA) within the past year. Sixty two subjects (50%) were observed to have evidence of one or more chronic diabetes complications.

Table 2 shows the results of comparison between the two groups, i.e. "better" and "poorer" groups using logistic regression. 48% of subjects who rated their health as very good or excellent exercised regularly, while it was a much smaller percentage in the "poorer" group (5.6%), with an OR of 15.69 which was highly significant (p = 0.003). As seen in table 2, there were no other significant differences between the groups.

**Table 2:** Group comparison

Variable	Poorer (%)	Better (%)	OR	CI	p value
**Female sex**	**55.6%**	**48%**	**1.35**	**0.40-4.57**	**.63**

**Regular clinic follow up (every 3 months)**	**72.2%**	**84%**	**2.02**	**0.46-8.92**	**.35**

**Intensive therapy (≥3 shots/day)**	**88.9%**	**92%**	**1.44**	**0.18-11.29**	**.73**

**SMBG (≥3 times/day)**	**83.3%**	**92%**	**2.30**	**0.34-15.44**	**.38**

**Regular exercise**	**5.6%**	**48%**	**15.69**	**1.80-136.62**	.003*

**Acute complications**	**44.4%**	**36%**	**.70**	**0.420-2.42**	**.58**

**Associated conditions**	**11.1%**	**16%**	**1.52**	**0.25-9.38**	**.65**

**Chronic complications**	**61.1%**	**36%**	**.36**	**0.10-1.25**	**.10**

**Positive family history**	**61.1%**	**64%**	**1.13**	**0.32-3.95**	**.85**

**Figure 2:**
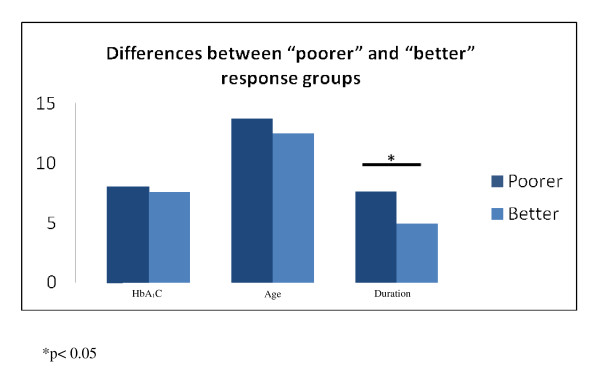
**Combined group comparison for age, diabetes duration and HbA**_**1**_**C**.

Figure 2 shows a comparison between the two groups in terms of diabetes duration, age and HbA_1_C values. The mean diabetes duration in the group labeled as "poorer" was significantly longer than that of those labeled as "better" health ( 7.62 ± 3.98 vs. 4.91 ± 3.45, *p *= 0.02). Whereas in terms of age, there was no statistically significant difference between the "poorer" and the "better" groups, respectively (13.72 ± 2.65 vs. 12.49 ± 3.29, *p *= 0.18). This was also seen in terms of differences in mean HbA_1_C values (poorer: 7.56 ± 1.09 vs. better: 8.02 ± 1.82, *p *= 0.34). An interaction effect between diabetes duration and regular exercise was tested for and there was no interaction found. After controlling for age, gender and diabetes duration, the regression model again showed that regular exercise was the only predictor that was independently and significantly associated with a "better" self-health rating, with an OR of 12.84, CI of 1.425-115.727 and a *p *value of 0.023.

## Discussion

Individuals with type 1 diabetes are challenged on a daily basis by the demands of diabetes management and the fear of complications; hence, their health related quality of life can be easily and considerably affected. In developed countries, subjects with T1DM have been shown to have a lower HRQoL than the general population [18,19], thus making it essential to include improvement of HRQoL in the management plan. This challenge becomes further pronounced in developing countries where quality of life is essentially compromised by the stress of meeting the basic needs of daily life. Since parents are the ones handling most financial stressors, and being the primary caregivers for younger children with diabetes, this stress can easily affect their children and diabetes care. Therefore, studying health related quality of life among young Egyptians with T1DM, especially of lower socio-economic status, and its relation to risk factors can help us understand and identify the best treatment regimen and target persons with a low HRQoL for intervention in order to alleviate their diabetes related daily stressor(s).

In our study, we attempted to compare the groups that either rated their health as better or poorer than children their age. We were interested in knowing what factors made these groups different. To our surprise, the only significant differences between the two combined groups were diabetes duration and presence/absence of regular exercise. Management regimen, sex, age and presence/absence of complications whether acute or chronic, were not different between the 2 groups. It is possible that no difference was found due to the study design or power; however, by employing regression analysis with a 95% confidence interval, we believe that our findings are to a good degree reliable. Similarly, presence of a positive family history, which could either influence the attitude towards diabetes in a positive or negative manner depending on this family's experience with the disease was not any different between the groups.

A weekly diabetes education program with 3-4 classes a week has been instituted at our hospital since about 6 years ago. Newly diagnosed children and their parents as well as non-compliant previously diagnosed children are required to attend these classes. Although an interaction effect between diabetes duration and exercise was not seen, a possible explanation for overall better health perception in our group of children who exercised regularly and had a shorter diabetes duration, would be that those who have been more recently diagnosed have been better educated and equipped with healthier diabetes management strategies, including regular exercise as a contributor to better long-term outcome of the disease. It may also be a sign that with the current diabetes epidemic, society has become more accepting of diabetes as a non-debilitating condition, perhaps encouraging those affected to participate in more sports activities and be more accepting of their condition as non-debilitating. In addition, Herman et al [1], in their study of prevalence of diabetes in Egypt and potential risk factors, have found that rural residents were least sedentary (52%), lower socioeconomic status urban residents were more sedentary (73%) and higher socioeconomic status urban residents were the most sedentary (89%). Our population consists mostly of the first two groups of residents, which could also explain the overall increased activity level among our subjects, but one that is mostly in the form of housework, farm work or long walks necessary to obtain daily basic needs.

A study by Tsai et al [11], looking at SRH among adults with T1DM showed that adults who reported being active had an increased likelihood of 81% for reporting excellent, very good or good SRH when compared with adults who reported being inactive regardless of diabetes status. Furthermore, they found that adults with diabetes who had ever taken a course or class (55.4%) for managing diabetes had a higher proportion of being active than diabetic patients who had never taken such a class (*p *< 0.001 for χ^2 ^test), which is in agreement with our results. Although, to our knowledge, exercise and SRH responses have not been looked at more closely in children with diabetes, studies have shown that those with a better glycemic control have a better health related quality of life, [20-22]. This again may be explained by the possibility that patients in those studies had been exercising more compared to the sample in the latter study. Exercise in developing countries is often regarded as a luxury. We try, however, to emphasize to our patients from the time of diagnosis that exercise is one of the main pillars in diabetes management. Mild to moderate exercise or activity is encouraged, even if it is in the form of housework or a walk to the furthest bus station or grocery store. Physical activity has been demonstrated to improve mental well-being [23]. In addition, regular exercise has been shown to improve blood glucose control, reduce cardiovascular risk factors, and contribute to weight loss [24,25]. The American Diabetes Association technical reviews on exercise in patients with diabetes, as well as other studies, have summarized the value of exercise in the diabetes management plan [25-27].

As regards to diabetes duration, studies have also shown either no association to diabetes duration [20,28] or a better health related quality of life with a shorter duration [29,30], which is similar to our results. Treatment regimen is another factor that has been looked at closely, again with studies showing either no association as in our study [28,31] or a better health related quality of life with more intensive treatment [18,32]. It is possible we did not see this association with more intensive treatment because the majority of our children are on the more intensive regimen, making any comparisons invalid. Other studies have shown that male gender [29,32], younger age [20,32], and higher socioeconomic status [12,20,30] were associated with better HRQoL, which again has not been the case in our study. This could imply either that there is variability in perception even with the same health status or that there are independent unmeasured risk factors for poor health in our population with type 1 diabetes.

Studies of adults with diabetes have consistently found that complications are associated with worsened health related quality of life [20,28,30]. Although complication rate was high among our patients (50%), it did not seem to affect SRH responses. This may be due to the fact that microalbuminuria and early retinopathy have no immediate impact on perception of health, but with the development of more overt kidney disease or vision problems as these children grow older, their health perception changes.

### Methodological considerations

Since our study subjects were volunteers and thus self-selected, there is a strong possibility of selection bias. Thus it is unclear the extent to which our findings are generalizable to other populations of children and adolescents with diabetes.

In addition, as is the case with any survey study, this study relies on a self-report method of data collection. A desire to please (interviewer/recall bias), poor memory, or misunderstandings of questions can all contribute to inaccuracies in the data. To minimize misunderstanding of questions, we have used an interviewer-conducted survey, which has the advantage of fewer misunderstood questions, fewer inappropriate responses and fewer incomplete responses, as well as generally higher response rates. Another potential limitation of our study is that we have not looked more closely at the exact parent income and education level and how they may affect health perception. However, as previously mentioned, the majority of our patients are of lower socioeconomic and education level. Questions on income and education level were therefore not asked since these are considered sensitive topics that could have discouraged our parents/patients from participating in the study.

Finally, we used self rated health as a measure of HRQoL in our study. It is fast, easy to obtain, and has been shown to be a powerful predictor of morbidity and mortality. Although it does not allow for the examination of risk factor impact on different aspects of HRQoL, as would other more complex measurements [18,22,28], nonetheless, we believe our results provide useful information for selecting potential risk factors, understanding correlations, and providing the basis for more detailed studies of HRQoL among young Egyptians with T1DM.

## Conclusions

In conclusion, metabolic control and health related quality of life are two important outcomes of T1DM care and need to be addressed in developing successful diabetes treatment strategies for children and adolescents with diabetes. Regular exercise is crucial to diabetes care and this study has further demonstrated a positive effect on the HRQoL of children with diabetes. Therefore, assessment and promotion of exercise and physical activity is important in achieving desired benefits with diabetes care.

## Abbreviations

T1DM: Type 1 diabetes mellitus; SRH: Self-rated health; HRQoL: Health- related quality of life; SMBG: Self monitoring of blood glucose; HbA_1_C: Glycohemoglobin; DKA: Diabetic ketoacidosis; OR: Odds ratio; CI: Confidence intervals.

## Competing interests

The author declares that they have no competing interests.

## Authors' contributions

HI has designed, collected and interpreted the data, as well as written the manuscript.

## Pre-publication history

The pre-publication history for this paper can be accessed here:

http://www.biomedcentral.com/1471-2458/11/216/prepub
